# Team Work among Nurses

**Published:** 1920-10-30

**Authors:** 


### THE MATRONS' AND SISTERS' DEPARTMENT.

### TEAM WORK AMONG NURSES.

While authorities are doubting and arguing which course of study is best for the health visitor, one or two keen observers are becoming aware that we are not attacking the nursing side of health problems in the best way. It is very curious to note how diversely we have set to work in this country to improve the health of our neighbours at the hands of nurses. The first impulse towards nursing the poor started with the Bible-women. Then came district nursing, and the effects of this mighty engine for good, reinforced by public sanitation, are already perceptible on a wide scale. Few homes to-day exhibit the hopeless degradation which was commonly presented to the eyes of the nurses thirty or forty years ago. Enormous strides have been made towards clearing away the remediable sufferings by which the poor in all ages have been afflicted. The entire district nursing movement has been initiated and developed under philanthropic endeavour. Like the voluntary hospitals, district nursing associations are the offspring of altruistic energy inspired by religion. They are among the most successful means men have been able to find towards the realisation of the truth that he who loves God must love his brother also.

From quite a different standpoint came the beginning of the health visitor movement. This originated in a high ideal of local government. It has been fostered by wide-minded men who recognised the responsibility of governing bodies for the well-being of the citizen, and obtained powers to enable them when necessary to tax the citizen himself in order to promote his health. Thus we are confronted with a dual system of organisation. The sick nurses are trained and maintained by voluntary agencies. The health nurses are paid and appointed by the people themselves through their representatives.

Since the county and district nursing associations have begun to receive subsidies from the rates, the difference between these two classes of nursing officials is perceptibly diminishing. They still continue, however, to work independently, and in certain districts there is evidence of very considerable friction between them. This friction is very seldom personal. It is purely administrative; that is to say, the authorities which define the duties of the nursing officials are liable to disagree about the lines of demarcation between them. The time seems to be approaching when some more centralised organisation may be arrived at.

All is tending towards a complete redistribution of the work accomplished by general, special, and health nurses. At present there is much overlapping, much waste of precious time, much waste of organising power, much quite futile routine work. The need is for co-ordinating, harmonising, vivifying, not for centralising in the sense of reducing all to a common official standard.

A central nursing organisation is called for in every town and in country areas of convenient size. At the head of it is needed a superintendent of comprehensive training and sympathetic insight into character, accustomed to deal with nurses and preferably with their training. From her would radiate, throughout the whole district entrusted to her care, nurses in various grades according to the dispositions made by the medical officers. All statistics would centre in her office, and in their preparation and the filing of reports and accumulation of records, of which the nurses would be relieved, V.A.D. help would be invited. All drugs, medical and surgical comforts, and appliances would be stored at the central office and be issued on signed requisitions. V.A.D. help would provide most of these and all surgical dressings. The staff would include fever, maternity, and general trained nurses, and if found desirable specialists also for school work and tuberculosis visiting. It would also be the centre for health visitors, and sanitary inspectors would find their duties greatly facilitated by the information made available for them and by the access to prompt aid and support when cases outside their own department confronted them, as must often happen. All the resources available for the combat of disease would be pooled and an immense accession of strength, no less than economy of administration, would result. The team would work in concert, all for one, one for all.

Such an ideal by no means presupposes the collapse of the voluntary organisations. They would still make themselves responsible for the upkeep of certain nurses, in special localities. But these would no longer work in isolation, exposed to the vicissitudes of fortune and cruelly fore-done in emergencies. They would form an honoured band in a united health service.

